# Insights for clinical management from the real-life data of the centralized West of Scotland biliary cancer clinic

**DOI:** 10.1186/s12885-024-12279-6

**Published:** 2024-05-16

**Authors:** Valentina Zanuso, Tamsin Nash, Raffaella Casolino, Gregory Armstrong, Ona Pallise, Jen Milne, Chiara Braconi

**Affiliations:** 1 School of Cancer Sciences, University of Glasgow, G61 1QH Glasgow, UK; 2 Department of Biomedical Sciences, Humanitas University, 20072 Pieve Emanuele, Milan, Italy; 3 Beatson West of Scotland Cancer Centre, G12 0YN Glasgow, UK; 4 NHS Greater Glasgow and Clyde, Glasgow, UK; 5 CRUK-Scotland Cancer Centre, Glasgow-Edinburgh, UK

**Keywords:** Cholangiocarcinoma, Biliary cancer, Genomic profiling, Systemic treatment, Chemotherapy, Scotland

## Abstract

**Background:**

With the increasing of novel therapeutics for the treatment of Biliary Tract Cancers (BTC), and the need to assess their socio-economic impacts for national licence approvals, it is as important as ever to have real-life data in national populations.

**Methods and results:**

We performed an audit of the first 2 year-activity (Sep 2019-Sep 2021) of the centralized West-of-Scotland-BTC clinic. 122 patients accessed the service, including 68% with cholangiocarcinoma (CCA), 27% with gallbladder cancer (GBC), and 5% with ampulla of Vater carcinoma with biliary phenotype (AVC). Median age at diagnosis was 66 (28–84), with 30% of newly diagnosed patients being younger than 60 years-old. Thirty-five cases (29%) underwent surgery, followed by adjuvant-chemotherapy in 66%. 60% had recurrent disease (80% with distant relapse). Sixty-four patients (58%) started first-line Systemic-AntiCancer-Treatment (SACT). Of these, 37% received second line SACT, the majority of which had iCCA and GBC. Thirty-% of those who progressed received third line SACT.

**Conclusions:**

About 30% of BTC were eligible for curative surgery. Fifty-eight and twenty% of the overall cohort of advanced BTC patients received first and second line SACT. Our data suggest that reflex genomic profiling may not be cost-effective until molecularly driven strategies are limited to second line setting.

**Supplementary Information:**

The online version contains supplementary material available at 10.1186/s12885-024-12279-6.

## Introduction

Biliary tract cancers (BTCs) are a group of rare and aggressive diseases whose incidence is increasing worldwide [1]. BTCs include cholangiocarcinoma (CCA) [subclassified as intrahepatic (iCCA), peri-hilar (pCCA) or distal (dCCA)], gallbladder cancer (GBC) and carcinoma of the Ampulla of Vater (AVC) [1]. Combined hepatocellular carcinoma (HCC) and CCA is another rare type of biliary cancer, associated with a dismal prognosis. Several controlled and randomized clinical trials have supported the benefit of additional drugs in selected populations of BTC patients, mainly focusing on targeted agents in the second-line setting [2, 3]. With the increase in novel therapeutic opportunities and the need for assessment of the socioeconomic impact of these novel strategies for national licence approvals, real-life data on the prevalence and disease course of BTCs in national populations becomes ever more useful. In addition, the need for genomic profiles as a pre-requisite for determining the eligibility for targeted drugs raises discussion around several issues, such as the need for tissue acquisition and the timeline for molecular testing in a cohort of patients who, unfortunately, decline more rapidly than most other cancer patients. The availability of real-life data enables the assessment of the proportion of patients who benefit from molecular testing to adopt cost-effective strategies that can be implemented at a governmental level. Since September 2019, the centralized West of Scotland (WoS) BTC clinic has been run from the Beatson Cancer Centre in Glasgow, with the aim of streamlining the pathway for BTC patients, converging their oncological treatment in a specialized centre and adopting a harmonized management with appropriate consideration for clinical trials and research projects. Taking advantage of this centralized approach, we have performed an audit of the activity of the WoS-BTC clinic for the first two years since its inception, allowing for a follow-up of 24 months, the timepoint at which 50% of tumour recurrence is expected after curative surgery and 90% of deaths are expected in the palliative setting [1].

## Methods

We performed a retrospective cohort analysis of BTC patients treated at the Beatson West of Scotland Cancer Centre from 2019 to 2021 under the Caldicott application entitled “Demographics and Clinical Characteristics of Patients with Biliary Tract Cancer in Scotland”, approved on 7/12/2021. The cohort consists of a repository of routinely collected clinical data. Clinical information was collected from electronic medical records and included demographic and tumour characteristics, type of treatment administered, and radiologic and outcome data. Clinical data were collected at different timepoints across treatment and follow-up and included data on diagnosis, type of treatment, disease progression and survival as determined by last follow-up date (October 2023). Patients seen at the WoS-BTC clinic (with medical and clinical (radiation) oncology input) from September 2019 to September 2021 were included in the cohort, irrespective of the year of diagnosis. All patients had a follow up of at least 2 years. All patients had been previously discussed at a specialized Multi-Disciplinary Team (MDT) meeting, where a referral to the oncology service was made for either discussing systemic anticancer treatment (SACT) in the context of a tissue diagnosis or assessing eligibility to SACT before acquisition of a tissue diagnosis in patients with borderline Performance Status (PS).

Recurrence Free Survival (RFS) was defined as the time from curative surgery to radiological relapse. Overall survival was defined as the time to death or last follow up from diagnosis or starting of first line SACT (as indicated each time). Scans were performed as per clinical practice guidelines, every 2–3 months, unless otherwise clinically indicated. Radiological Responses were assessed by radiologists according to RECISTS.1 criteria.

Descriptive statistical analyses have been performed through GraphPad Prisms (version 10.0).

This study was approved by the ethical Caldicott committee for NHS Greater Glasgow & Clyde on 7/12/2021 (Demographics and Clinical Characteristics of Patients with Biliary Tract Cancer in Scotland), and was given the approval for clinical audit and publication. No identifiable information are present in this manuscript as per application. Informed consent was not obtained as per Sect. 60 of the Health and Social Care Act 2001, given this is a retrospective study and does not contain identifiable information.

## Results

**Fig. 1:**
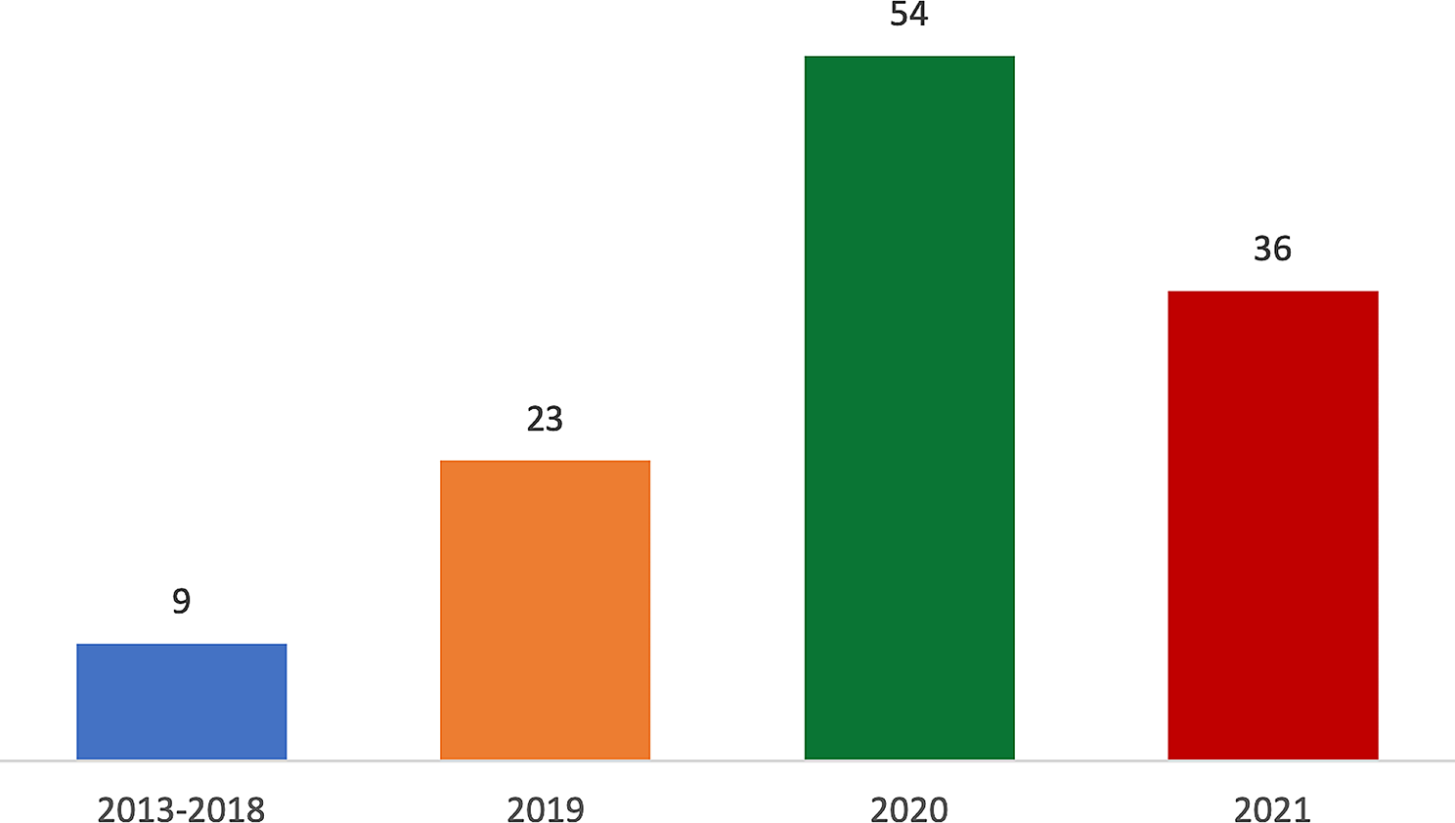
Distribution of new diagnoses per year in the population of the WoS-BTC clinic between September 2019 and September 2021. Abbreviations: BTC, biliary tract cancers

**Fig. 2:**
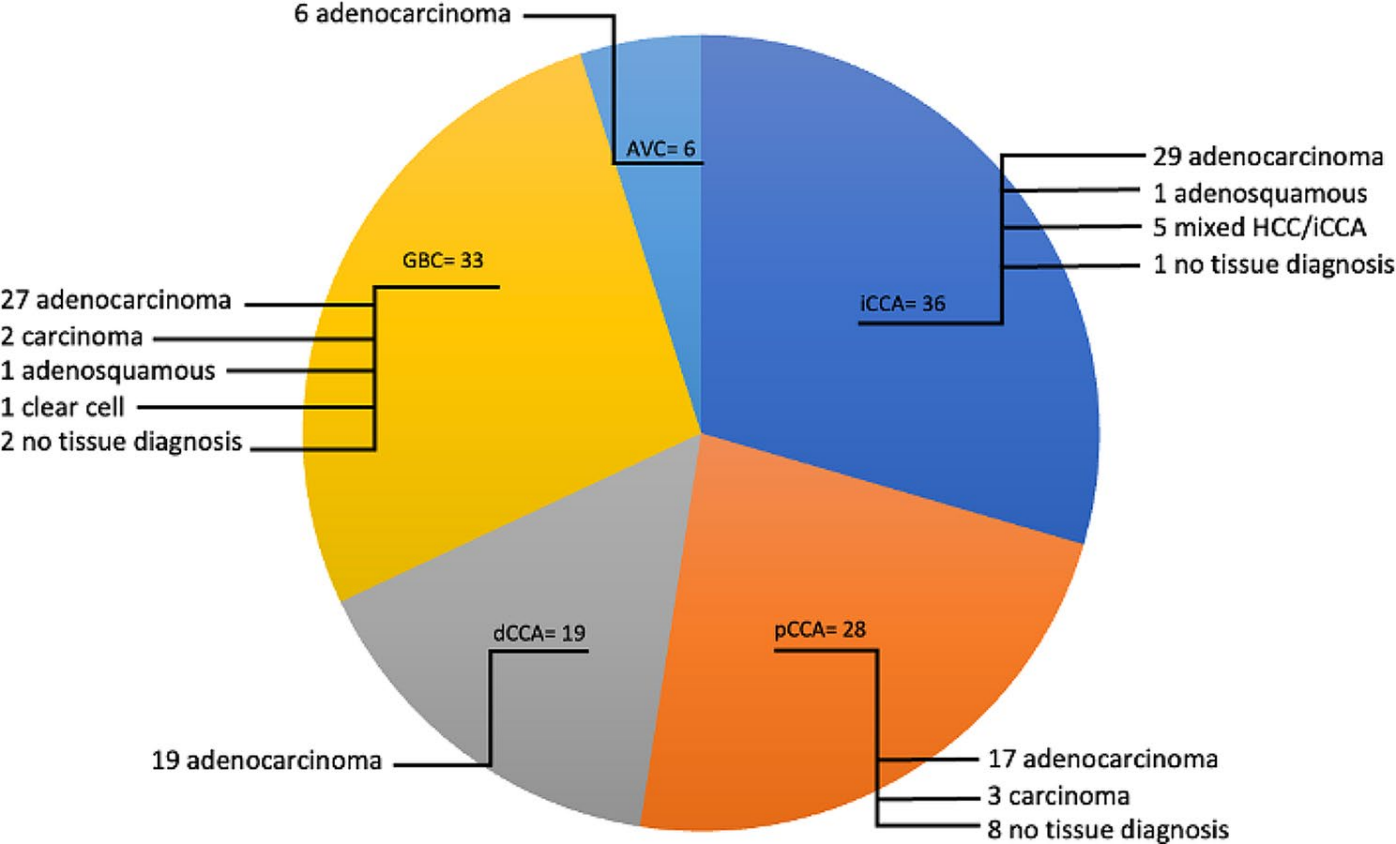
Histology according to BTC subtype. Abbreviations: BTC, biliary tract cancers; iCCA, intrahepatic cholangiocarcinoma; pCCA, perihilar cholangiocarcinoma; dCCA, distal cholangiocarcinoma; GBC, gallbladder cancer; AVC, carcinoma of the Ampulla of Vater

A total of 122 patients attended the WoS-BTC clinic between September 2019 and September 2021, with a diagnosis of BTC made between November 2013 and August 2021 (Fig. 1). Median follow up time from date of diagnosis was 12.98 months. Sixty-four (52%) and 58 patients (48%) were female and male, respectively. Median age at diagnosis was 66 years (range 28–84), with the following distribution per age group: <40 (3 [2%]); 40–59 (32 [26%]); 60–79 (82 [67%]); ≥80 (5 [4%]). The cohort included 36 iCCA patients (30%; with 5 mixed HCC/iCCA), 28 pCCA patients (23%), 19 dCCA patients (15%), 33 GBC patients (27%) and 6 AVC patients (5%) (Fig. 2). Tissue diagnosis was not achieved in 11 patients (9%), either because of poor Eastern Cooperative Oncology Group (ECOG) PS (*n* = 8) or repeated unsuccessful biopsy attempts (*n* = 3) (Suppl Table 1). Of patients without tissue diagnosis, 73% had a radiological diagnosis of pCCA and of these, 6 out of 8 patients did not proceed to further active treatment.

**Fig. 3:**
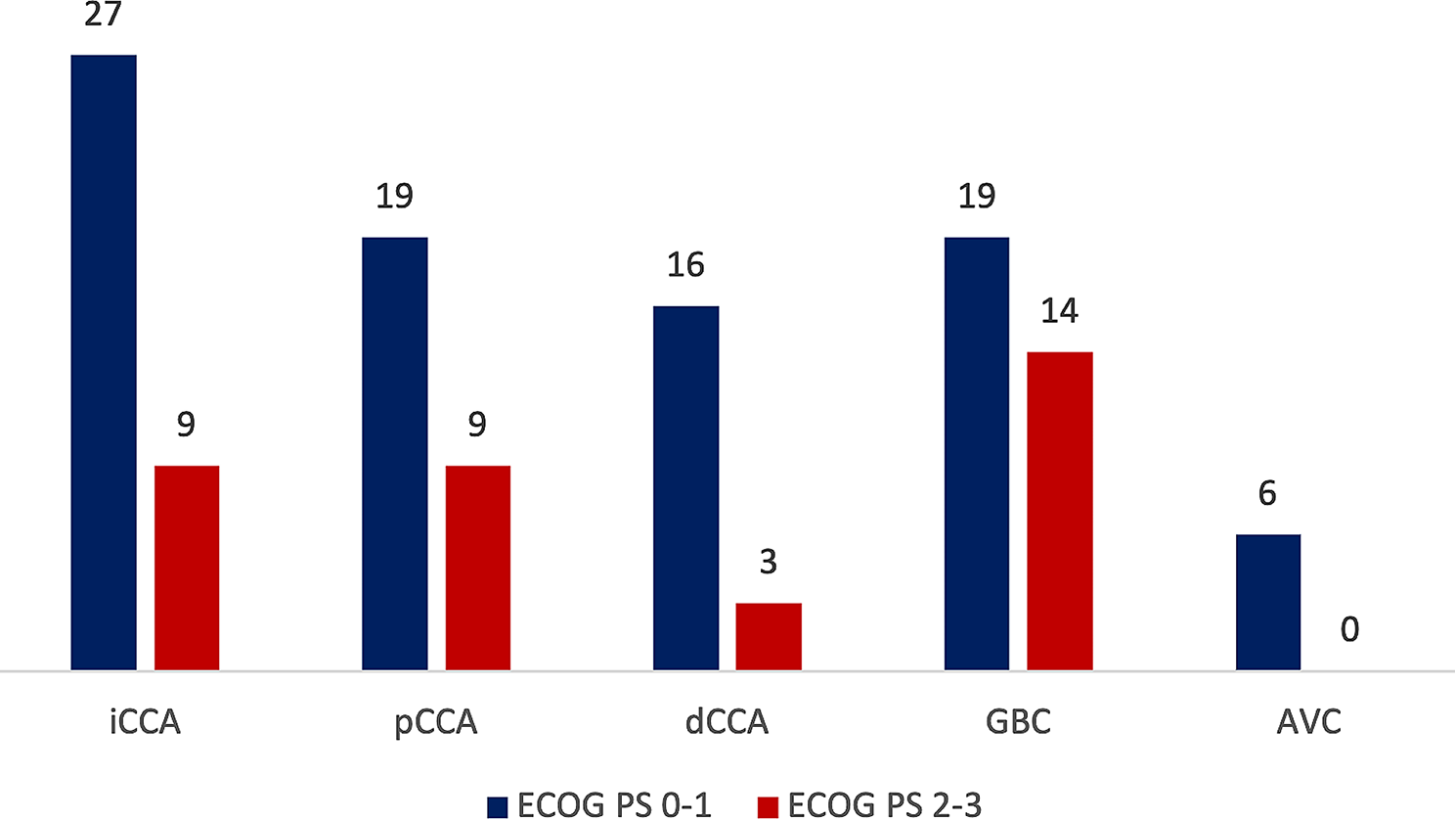
ECOG PS at BTC diagnosis. Abbreviations: ECOG, Eastern Cooperative Oncology Group; PS, performance status; BTC, biliary tract cancers; iCCA, intrahepatic cholangiocarcinoma; pCCA, perihilar cholangiocarcinoma; dCCA, distal cholangiocarcinoma; GBC, gallbladder cancer; AVC, carcinoma of the Ampulla of Vater

At first evaluation at the Beatson West of Scotland Cancer Centre, 71% of patients presented with a good ECOG PS (0–1) (iCCA 31%, pCCA 22%, dCCA 18%, GBC 22%, AVC 7%), whereas 35 patients presented with a poor ECOG PS (2–3), the majority of whom had a diagnosis of GBC (iCCA 25%, pCCA 25%, dCCA 10%, GBC 40%) (Fig. 3).

Seven patients (6%) presented with resectable disease but were inoperable due to comorbidities. This subgroup of patients had a median age of 70 years (range 44–80) and included 5 patients with iCCA, 1 patient with pCCA and 1 patient with dCCA.

At the time of diagnosis, 35 patients (29%) underwent surgery with curative intent, including hepatectomy (23%), Whipple surgery (37%), cholecystectomy (37%) and liver transplant (3%). Incidental diagnosis of cancer in surgical specimens was noted in 20% of cases, including 6 cases of GBC at cholecystectomy performed for suspected cholecystitis and 1 case of pCCA at liver transplantation performed for Primary Sclerosing Cholangitis (PSC). Negative resection margins (R_0_) were achieved in 21 patients (60%). Lymph nodal metastases were detected in 15 cases (43%), of which 40% were also R_1_ (tumour within 1 mm of resection margins), while 93% had lymphovascular invasion detected microscopically. Perineural and lymphovascular invasion were found in 25 (71%) and 18 patients (51%), respectively, with 16 patients (46%) having both. Adjuvant chemotherapy was administered in 23 patients (66%); the most common treatment regimen was capecitabine (18 patients, 78%), followed by cisplatin plus gemcitabine (2 patients, 9%), gemcitabine plus capecitabine (2 patients, 9%) and gemcitabine monotherapy (1 patient, 4%). Twelve patients (34%) did not receive adjuvant chemotherapy: 5 patients were treated before 2019 (evidence from the BILCAP study [4]); 2 patients had an early relapse and received palliative SACT; 5 patients had post-operative complications which resolved beyond 12 weeks from surgery. With a median follow-up from surgery of 44.0 months, 21 patients (60%) experienced disease recurrence, of whom 12 (57%) received adjuvant SACT. None of the patients received adjuvant radiotherapy. Only 4 patients had local recurrence in the absence of metastatic disease (2 patients with R_0_ resection margins and 2 patients with R_1_ resection margins; 3 of these patients had N + disease) (Suppl Figs. 1 and 2).

**Fig. 4:**
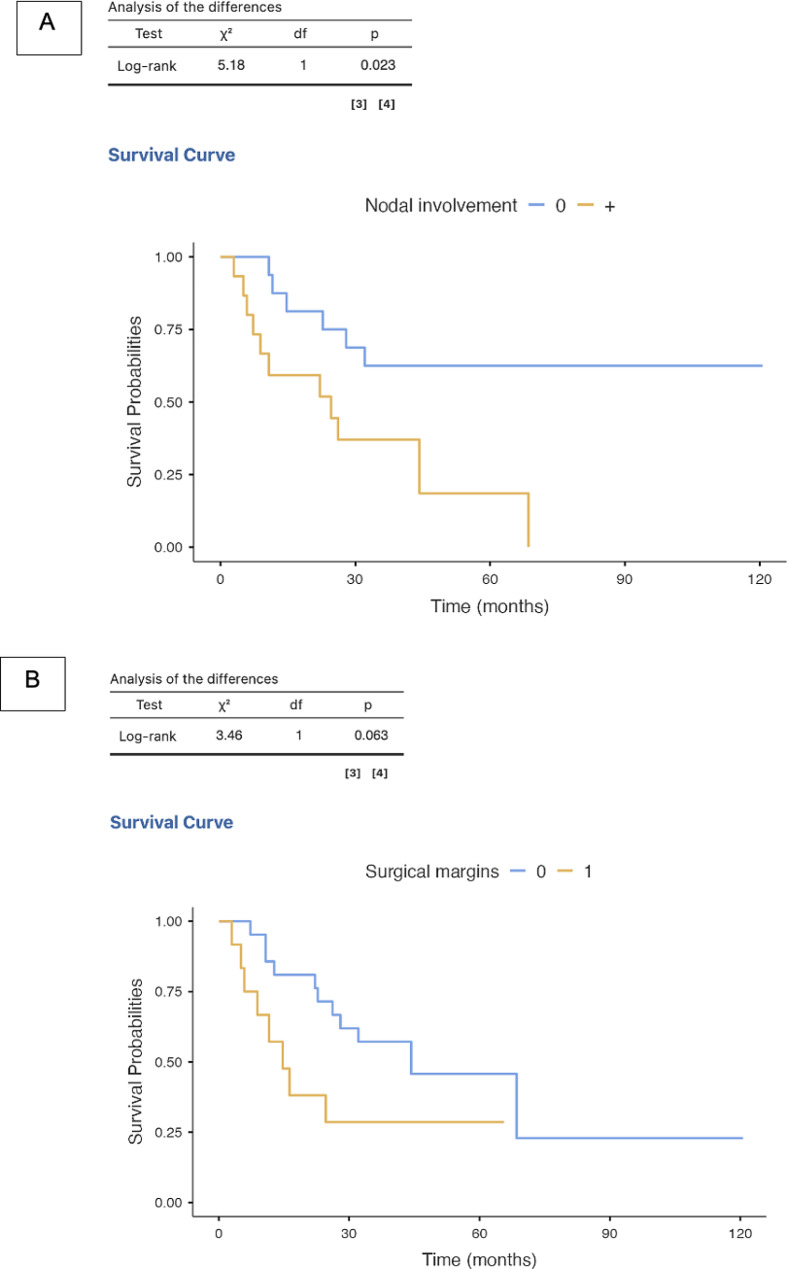
Median time to recurrence according to nodal status (**A**) and status of surgical margins (**B**)

Almost half of the patients who underwent curative surgery (49%) developed metastatic disease at some stage, mainly involving liver and peritoneum (33% R_1_, 33% N+, 22% both R_1_ and N+). Patients with positive lymph nodes had a higher risk of recurrence compared to negative ones (*p* = 0.023) (Fig. 4A), whereas there was no statistically significant difference between R_0_ and R_1_ patients (*p* = 0.063) (Fig. 4B). The median time from curative surgery to disease recurrence was 16.3 months in the whole cohort (8.0 months for local recurrence only, 22.8 months for metastatic disease). Out of 21 patients, 14 (67%) proceeded to SACT (iCCA 8%, pCCA 21%, dCC 14%, GBC 43%, AVC 14%).

Eight-seven patients (71%) were diagnosed with locally advanced or metastatic disease at diagnosis. The most common sites of metastasis were liver, nodes and peritoneum.

Among patients diagnosed with non-resectable, recurrent and/or locally advanced/metastatic disease, 41 patients (38%) received best supportive care (BSC, including stenting and symptoms control provided by general practitioners), and 2 patients (2%) locoregional treatment (1 surgery and 1 radiofrequency ablation [RFA]). Amongst those deemed unfit for SACT, median age was 68 years old (range 39–84) and the majority included GBC (39%) and iCCA (27%). Median Overall Survival (OS) in this group was 4.7 months (95% CI, 3.9–8.1). Sixty-four patients (58%) received first-line SACT: the majority of patients (45, 70%) received a combination of platinum-based therapy, followed by gemcitabine monotherapy (14, 22%). 18 patients (28%) were enrolled in first line SACT clinical trials. Median duration of SACT was 2.5 months [range: 0.2–14.7]. For those who interrupted SACT before 5 months (*N* = 49), the majority stopped because of radiological PD or clinical deterioration (70%), 14% because of risk/benefit assessment during the pandemics, 6% because of personal patient’s choice, 6% because they underwent surgical assessment and 4% because of toxicity, Sixty-three out of 64 patients experienced disease progression, with a median time to disease progression of 6.7 months. Among progressed patients, 31 patients (49%) were treated with best supportive care (BSC), whereas only 23 patients (37%) were eligible for a second-line systemic treatment, mainly represented by FOLFOX or CAPOX. Overall, only 20% of the overall cohort received second-line SACT– these patients included mainly iCCA (35%), followed by GBC (26%), pCCA (23%), dCCA (17%). All second-line-treated patients progressed, however, only 7 of them (30%) received another line of systemic treatment.

**Fig. 5:**
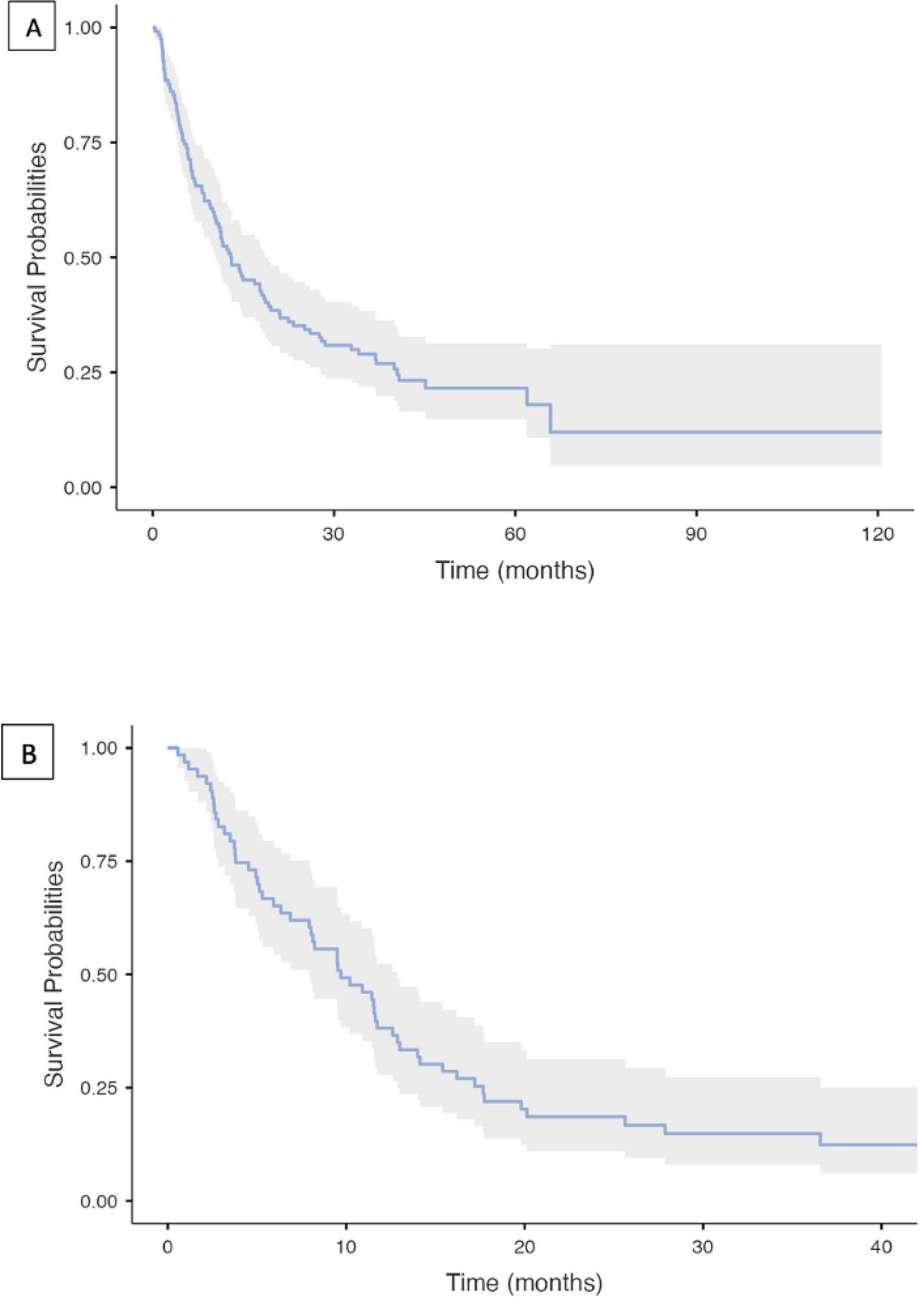
Overall survival from diagnosis (**A**) and from beginning of first line (**B**)

At the last follow-up, 94 patients (77%) were dead. In the overall population, median OS from diagnosis was 13.0 months (95% CI, 10.5–18.6) (Fig. 5). In the resected cohort, median OS was 61.9 months (95% CI, 40-not reached [NR]), while OS at 2 years was 83%. Among patients receiving first-line treatment, median OS from diagnosis was 14.5 months (95% CI, 11.6–19.2), whereas median OS from start of systemic treatment was 9.7 months (95% CI, 7.9–12.9) (Fig. 5), with 2-year OS of 19%.

**Fig. 6:**
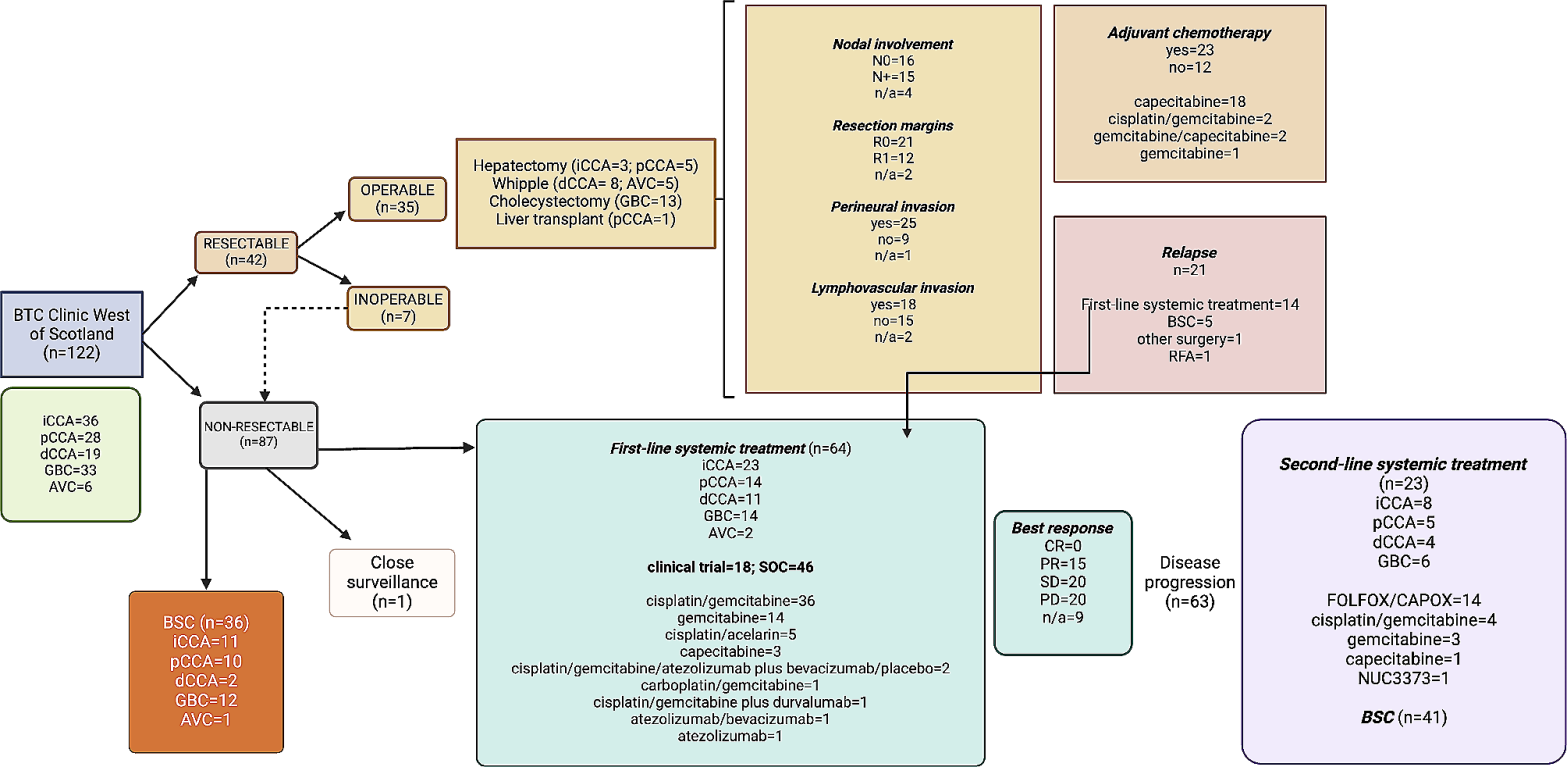
Management of BTC at the Beatson West of Scotland Cancer Centre from 2019 to 2021. Abbreviations: BTC, biliary tract cancers; iCCA, intrahepatic cholangiocarcinoma; pCCA, perihilar cholangiocarcinoma; dCCA, distal cholangiocarcinoma; GBC, gallbladder cancer; AVC, carcinoma of the Ampulla of Vater; BSC, best supportive care; SOC, standard of care; CR, complete response; PR, partial response; SD, stable disease; PD, progressive disease; n/a, not available

Overall summary of the cohort is represented in Fig. 6 and Suppl Figs. 3–6. Figure 6 summarises the management of our cohort of BTCs.

## Discussion and conclusion

Incidence of BTC continues to increase worldwide. Recent English epidemiological data have shown that the number of cases of iCCA equals those of HCC, making BTC a growing social problem in the UK [5]. This issue is worsened by our observation that 30% of newly diagnosed patients are younger than 60 years old, suggesting these diagnoses will also have a social impact on the workforce of the country. In our experience, we noticed that the centralization of management of BTCs led to an increase in the awareness of this disease, with optimization of oncological referrals and involvement of patients in research. We have witnessed over the last ten years an exciting revolution within therapeutic strategies for treatment of BTCs, with several drugs that have received scientific confirmation of their activity and have therefore been taken to regulatory bodies for approval in clinical practice [6–17]. In Scotland there is still a limited access to targeted therapies, with only Pemigatinib being approved since February 2022. However, during discussion of the cost/benefit ratio of these new approaches, some data may yet be absent, either because the ultra-selection of patients does not allow phase III randomized trials or because new standards of care have been introduced, changing the comparator arms. In this scenario, real-life data gather is critical to understanding the distribution and outcome of the disease in a specific population. Here, we report the activity of the centralized WoS-BTC clinic. Despite this being a small, single-centre study, it has the advantage of: (1) allowing the consideration of patients converging from different referral hospitals, (2) reflecting the current status of the West of Scotland, (3) not being biased by the expertise of the centre as it converges all BTC patients considered in the region for SACT independently of surgical expertise, (4) not including out-of-region referrals. A total of 122 patients have accessed the WoS-BTC clinic over a period of two years, with a predominance of iCCA (29%) and GBC (27%), suggesting that patients with these subtypes of BTC are more likely to undergo SACT. Indeed, iCCA patients represented 31% of those presenting with good PS (ECOG 0–1), for whom SACT was recommended. pCCA is known to represent a challenging disease and discussion is still ongoing in the medical community on the best approach for these patients, as biliary drainage is often difficult to achieve. We have observed a lack of tissue confirmation in 9% of all our pCCA underlining the difficulties of reaching a proper diagnosis– especially after insertion of a metal stent– which bolsters the argument for a centralised discussion of these patients before a plan is implemented. Nonetheless, most of these patients were deemed not fit for SACT, underlining that an appropriate discussion can save resources for the system and ineffective and invasive procedures for the patient.

Our data are overall in line with that reported in clinical trials and larger real-life data generated though international registry [18], with 30% of BTC patients diagnosed with early-stage disease amenable to curative treatment. Patients diagnosed with advanced BTC had a median OS of about 10 months, slightly lower than the 11.7 months observed in the ABC-02 study, likely related to less stringent criteria for recommendation of SACT in clinical practice in comparison to a phase 3 trial. Nonetheless the 2 years OS rate was 19% in our cohort in comparison to the 11% reported in the literature, confirming that BTC is a heterogenous disease, and a small niche of patients can have a better prognosis and derive better benefit from SACT [19, 20].

29% of the whole cohort of BTC patients were eligible for curative surgery, of which 60% relapsed within 2 years. We confirmed that the most accurate prognostic factor for relapse was the lymph-nodal status, as previously reported [21]. Interestingly, we did observe a high correlation between positive nodal status and microscopic vascular invasion (93%), suggesting that in those cases where assessment of lymphoadenectomy is considered (i.e. incidental diagnosis of GBC), the presence/absence of microscopic vascular invasion in the pathological specimen could add useful information in guiding the treatment choice. It is also of note that in a cohort where 60% of patients relapsed after surgery for BTC, only 11% presented with local recurrence in absence of distant metastases (irrespective of the status of resection margins), and even in these cases 75% of tumours were N+. Altogether, these data highlight that the recurrence of BTC is driven from dissemination of the disease, making the case for neoadjuvant SACT. Moreover, a neoadjuvant approach would also provide an opportunity for better control of the disease in about 15% of patients who miss the window of opportunity for adjuvant chemotherapy due to post-surgical complications. We do acknowledge the risks associated with a neoadjuvant approach, specifically (1) side effects from SACT could deteriorate the fitness of the patients and preclude curative intervention, which prompt a careful assessment of the regimen considered, especially in the era of immunotherapy when the immune related side effects can have a delayed onset and be long term; (2) there is 20–30% of risk of progressive disease before surgery is considered. However, this outcome usually reflects an aggressive biology and a lack of clinical benefit from surgery as these patients often quickly progress with metastatic disease that was present but occult at diagnosis, Adjuvant radiotherapy was not recommended in our practice given the lack of phase 3 randomized data; the benefit suggested from previous metanalysis in R1 cases may however be limited given the low rate of isolated local recurrences we have observed [22].

In our cohort, 58% of advanced BTC patients received first-line treatment, of which 31% were enrolled in clinical trials. Progression to first line has occurred in 98% of cases, of which 37% proceeded to second-line systemic treatment, representing 20% of the whole cohort.

With the introduction of targeted therapies in the second-line setting of BTC patients, molecular profile has become mandatory. However, the timing for the undertaking of genomic testing is still debatable given declining PS may prevent access to second-line therapies if the procedure is initiated at progression to first line. The option of reflex testing, triggered by the pathological diagnosis without considering other variables, has been considered. According to our data, however, this may not represent the best cost-effectivene strategy. If we consider resected samples from curative surgery (30% of the whole cohort), only 40% of these patients initiate palliative SACT, suggesting that overall, the genomic profile would not add information at the present time in at least 60% of these cases. When considering non-resectable patients, pathological diagnosis of BTC may not be associated with active SACT, as shown by our experience where 38% of patients underwent best supportive care due to reduced fitness. Despite only 37% of patients starting first-line SACT proceeded to a second line (where all targeted therapies are currently indicated), initiating genomic profiling at the beginning of first line may represent an acceptable compromise to optimise the resources of the health system, while assuring the best treatment for patients.

Several are the limitations of this study. It suffers from a limited sample size, does not provide information on the outcome of patients undergoing immunotherapy in combination to cisplatin and gemcitabine, and it includes patients treated during the severe acute respiratory syndrome coronavirus 2 (SARS‑CoV‑2) pandemics where the health system in Scotland had undergone rearrangement of its activity with impact on the organisation of surgical lists and delivery of oncological treatments. In addition, this cohort does not reflect the totality of the West of Scotland population of BTC patients, as it excludes patients who were deemed not fit for SACT during MDT discussion. Nonetheless, these data provide information about the natural distribution of BTC subtypes and associated outcomes, and provide useful insights for the implementation of novel therapeutic strategies in this Scottish population, many of which can be expanded to wider populations.

### Electronic supplementary material

Below is the link to the electronic supplementary material.

Supplementary Material 1

## Data Availability

The datasets used and/or analysed during the current study are available from the corresponding author on reasonable request.

## Abbreviations

BTC: Biliary Tract Cancer
CCA: Cholangiocarcinoma
iCCA: Intrahepatic CCA
pCCA: Perihilar CCA
dCCA: Distal CCA
GBC: Gallbladder Cancer
AVC: Ampulla of Vater Cancer
SACT: Systemic AntiCancer Therapy
HCC: Hepatocellular Carcinoma
WoS: West of Scotland
MDT: MultiDisciplinary Team
PS: Performance Status
RFS: Relapse Free Survival
OS: Overall Survival
PSC: Primary Sclerosing Cholangytis
BSC: Best Supportive Care
RECIST: Response evaluation criteria in solid tumors
