# Supplementary material for: Insights for clinical management from the real-life data of the centralized West of Scotland biliary cancer clinic
Source: BMC Cancer. 2024 May 16;24:597. doi: 10.1186/s12885-024-12279-6 (PMC11097428; doi:10.1186/s12885-024-12279-6)
Supplement: Supplementary file 1 — Supplementary Material 1 [file 12885_2024_12279_MOESM1_ESM.pdf]

Suppl Fig 1. Distribution of Time to Recurrence (TTR) for patients undergoing curative surgery according to the status of resection margins. Each column represents one patient.

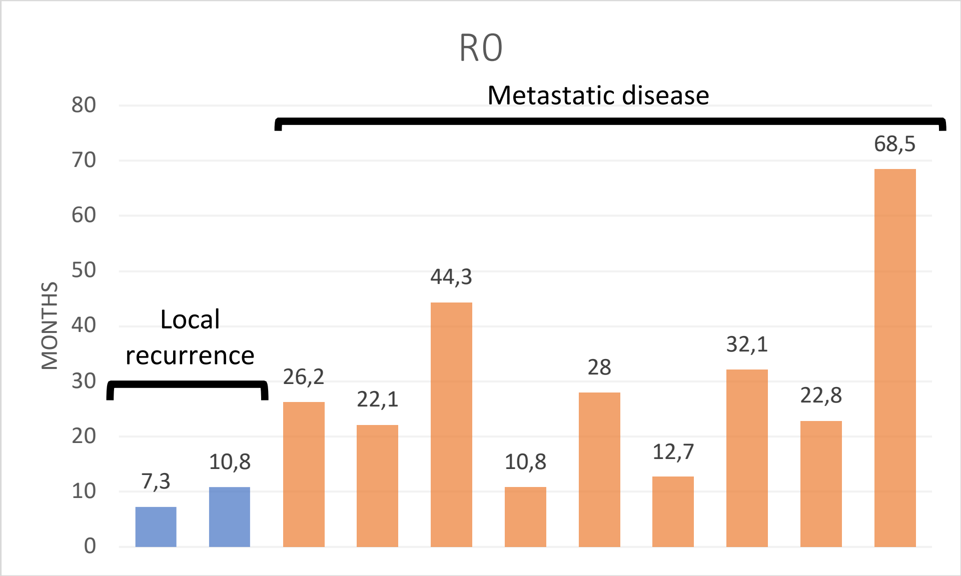

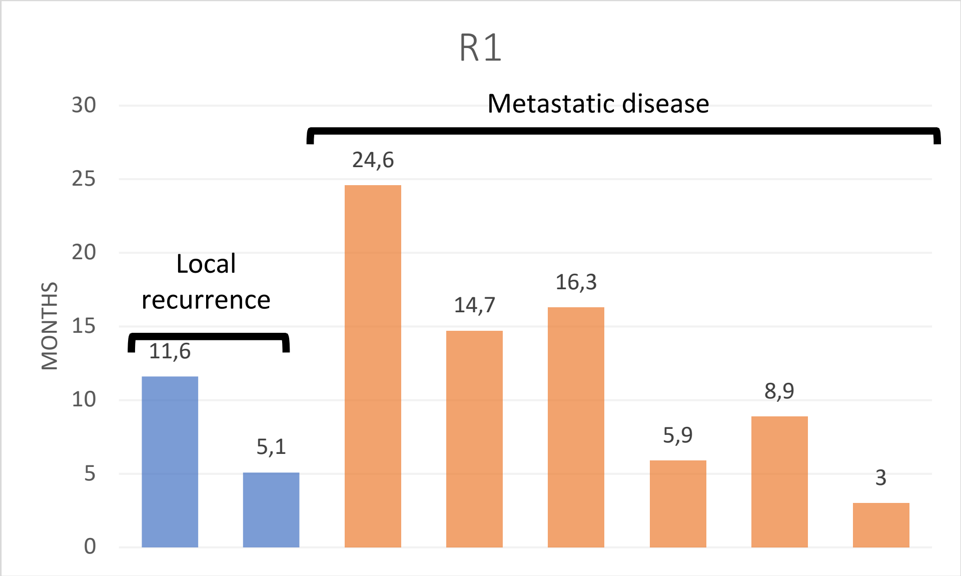

Suppl Fig 2. Distribution of Time to Recurrence (TTR) for patients undergoing curative surgery according to the nodal status. Each column represents one patient.

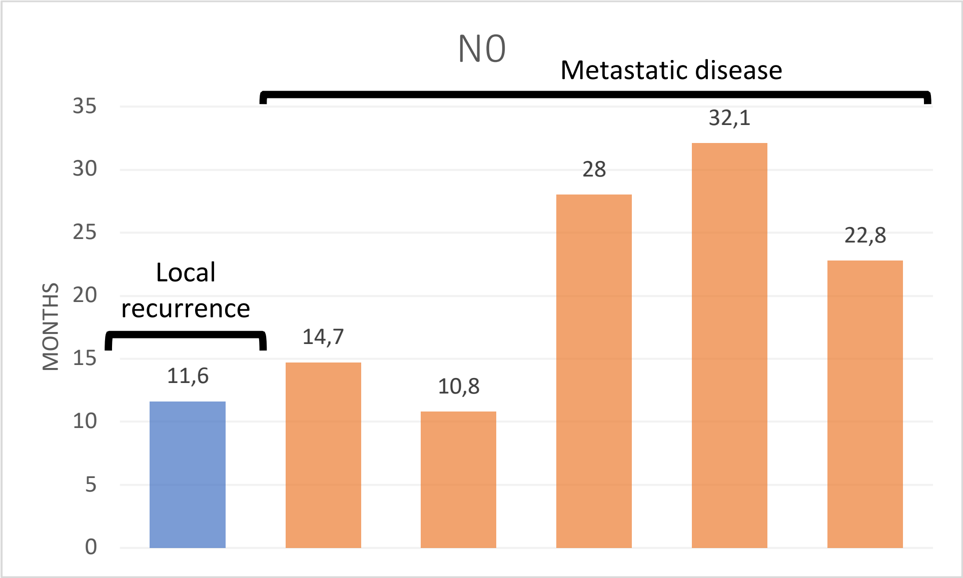

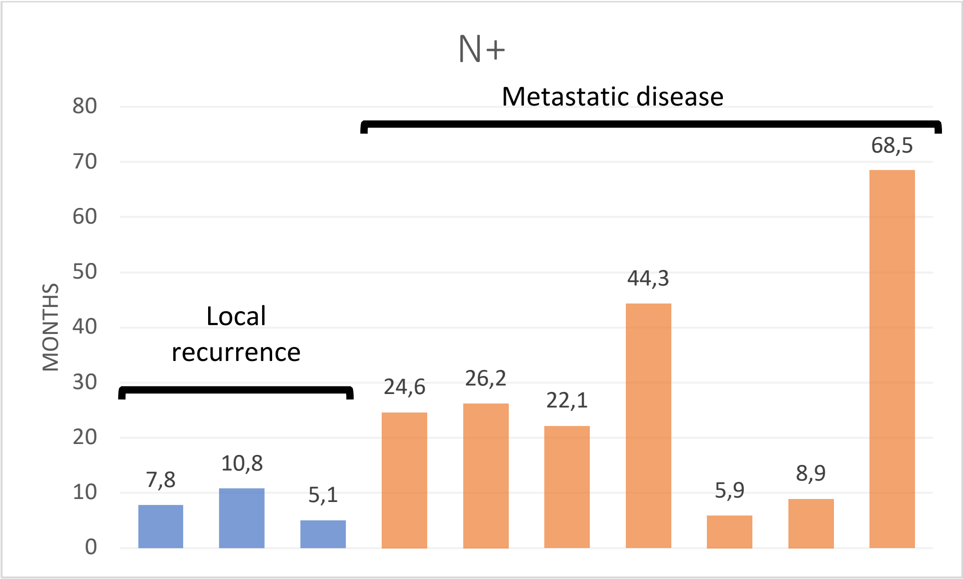

Suppl Figure 3. Management of iCCA at the Beatson West of Scotland Cancer Centre.

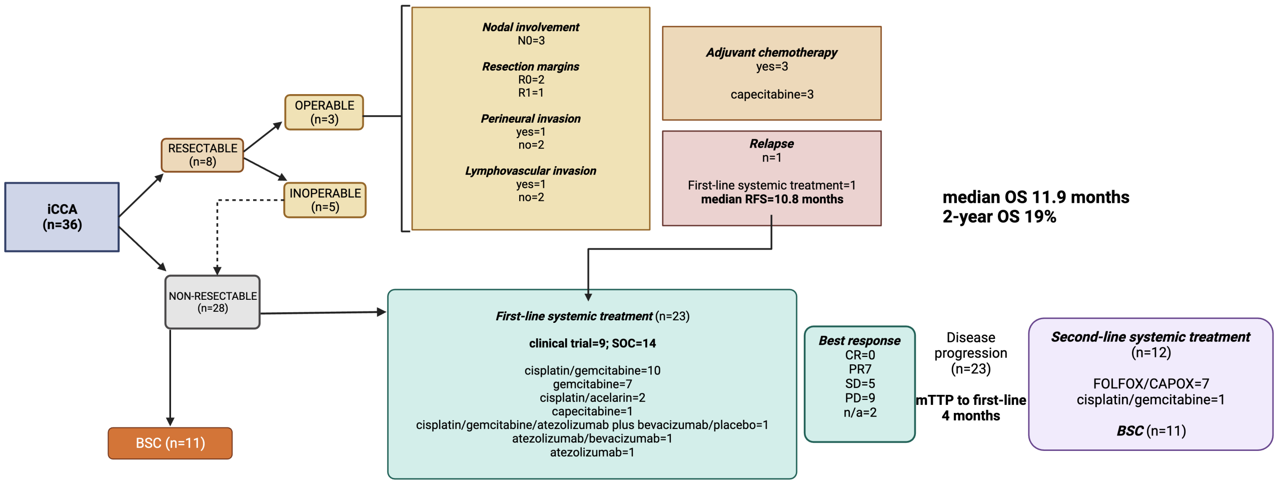

Abbreviations: iCCA, intrahepatic cholangiocarcinoma; BSC, best supportive care; SOC, standard of care; CR, complete response; PR, partial response; SD, stable disease; PD, progressive disease; n/a, not available; RFS, recurrence-free survival; OS, overall survival; mTTP, median time to progression.

Suppl Figure 4. Management of pCCA at the Beatson West of Scotland Cancer Centre.

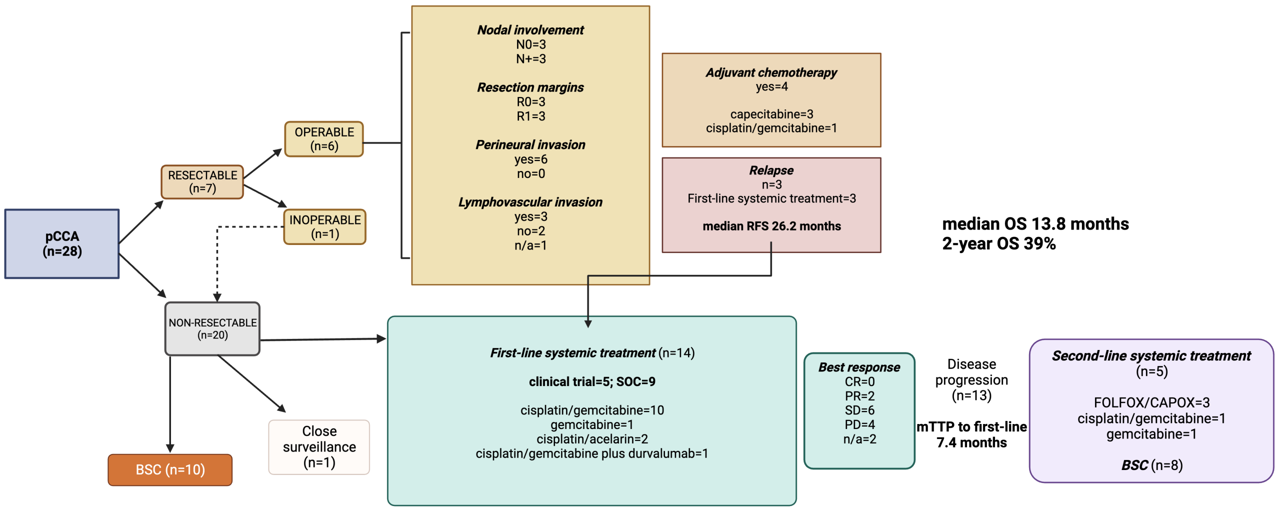

Abbreviations: pCCA, peri-hilar cholangiocarcinoma; BSC, best supportive care; SOC, standard of care; CR, complete response; PR, partial response; SD, stable disease; PD, progressive disease; n/a, not available; RFS, recurrence-free survival; OS, overall survival; mTTP, median time to progression.

Suppl Figure 5. Management of dCCA at the Beatson West of Scotland Cancer Centre.

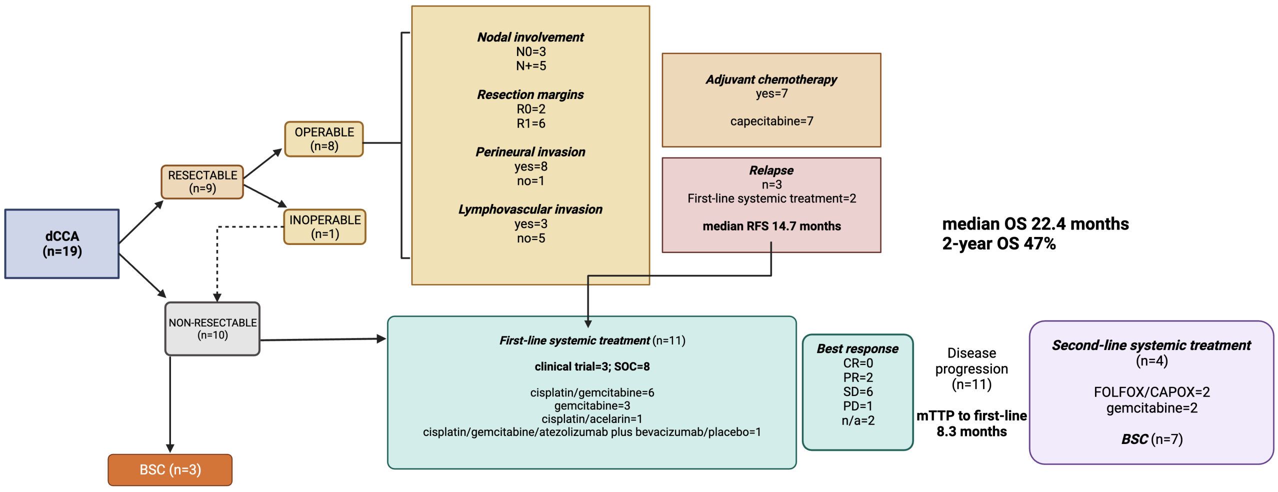

Abbreviations: dCCA, distal cholangiocarcinoma; BSC, best supportive care; SOC, standard of care; CR, complete response; PR, partial response; SD, stable disease; PD, progressive disease; n/a, not available; RFS, recurrence-free survival; OS, overall survival; mTTP, median time to progression.

Suppl Figure 6. Management of GBC at the Beatson West of Scotland Cancer Centre.

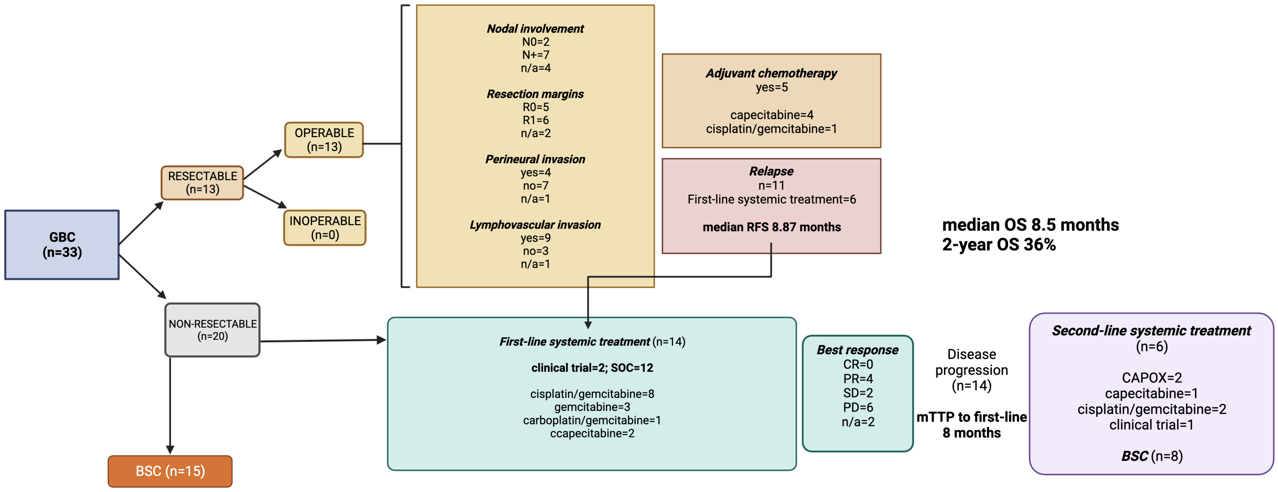

Abbreviations: GBC, gallbladder cancer; BSC, best supportive care; SOC, standard of care; CR, complete response; PR, partial response; SD, stable disease; PD, progressive disease; n/a, not available; RFS, recurrence-free survival; OS, overall survival; mTTP, median time to progression.

Suppl Table 1. BTC without tissue diagnosis.

No tissue diagnosis		
iCCA	1	poor ECOG PS
pCCA	8	2 unsuccessful biopsy<br>6 poor ECOG PS
GBC	2	poor ECOG PS

Abbreviations: BTC, biliary tract cancers; iCCA, intrahepatic cholangiocarcinoma; pCCA, peri-hilar cholangiocarcinoma; GBC, gallbladder cancer; ECOG, Eastern Cooperative Oncology Group; PS, performance status.
